# Coexisting frailty and depression associated with low physical activity and quality of life in Saudi community-dwelling older adults: a cross-sectional study

**DOI:** 10.3389/fpubh.2025.1531101

**Published:** 2025-05-23

**Authors:** Norah A. Alhwoaimel, Bader A. Alqahtani, Mohammed M. Alshehri, Ahmed S. Alhowimel, Aqeel M. Alenazi

**Affiliations:** ^1^Department of Health and Rehabilitation Sciences, Prince Sattam Bin Abdulaziz University, Al-Kharj, Saudi Arabia; ^2^Department of Physical Therapy, Jazan University, Jazan, Saudi Arabia

**Keywords:** frailty, depression, physical activity, quality of life, older adult

## Abstract

**Background:**

Frailty and depression are common conditions in older adults, but their coexistence and combined impact on physical activity and quality of life remain understudied, particularly in Saudi Arabia.

**Objective:**

To examine the independent associations of depression, frailty, and their coexistence with physical activity levels and quality of life among Saudi older adults.

**Methods:**

A cross-sectional study was conducted among 395 community-dwelling adults aged 60 years and older (mean age 66.4 ± 7.1 years, 60% women) in Saudi Arabia. Frailty was assessed using the FRAIL scale, depression using the Patient Health Questionnaire 9 (PHQ-9), physical activity using the Physical Activity Scale for the Elderly (PASE), and quality of life using the SF-12. Participants were categorized based on the results of FRAIL and PHQ-9 scale into four groups: coexisting frailty and depression, frailty only, depression only, or neither.

**Results:**

The prevalence of coexisting frailty and depression was 8.6%. Participants with coexisting frailty and depression were more likely to be sedentary (OR = 4.37, 95% CI: 1.41–13.55, *p* = 0.011) compared to those without frailty or depression. Frailty only group (*β* = −3.55, *p* = 0.003) and coexisting frailty and depression (*β* = −2.55, *p* = 0.033) were significantly associated with lower physical quality of life. Depression, frailty, and their coexistence were all significantly associated with lower mental quality of life (*p* < 0.001 for all).

**Conclusion:**

The coexistence of frailty and depression in older adults is associated with a higher likelihood of sedentary behavior and lower quality of life. These findings highlight the need for comprehensive geriatric assessments and targeted interventions addressing both physical and mental health in older adults.

## Introduction

The aging population is rapidly growing worldwide. Approximately 703 million people were aged 65 years and older in 2019, and this figure is predicted to grow substantially to about 1.5 billion by 2050 (1). In Saudi Arabia, the older population is expected to rise from the 9.5% in 2035 to 18% of the entire population by the year 2050.

The aging process, a complex phenomenon involves psychological, biochemical, and functional changes that cause a decline in physiological and functional capacities, leading to frailty. Frailty is increasingly among older adults, with more than half of the older adult population in Saudi Arabia experiencing some degree of this condition (1). A recent cross-sectional study reported that 21.4% of older adults in Riyadh were frail, while 47.3% were pre-frail (1). The key factors independently associated with frailty among Saudi older adult include age, living alone, cognitive impairment, and multimorbidity (1). The prevalence of frailty was significantly (*p* < 0.001) associated with falls among Saudi older adults and frail older adult have 5.37 times higher number of falls compared with non-frail individuals (2). Frailty increases vulnerability to negative health outcomes, such as fatigue, weakness, disability, falls, hospitalization, imposing significant healthcare burdens and contributing to depression (3, 4).

Depression a major mental health issue among older adults and is often linked to physical decline, cognitive impairment, and reduced quality of life (QoL) (5). Recent studies found that 42.4% of older adults aged 60 years and older in Saudi Arabia exhibited depressive symptoms (6). The prevalence of depressive symptoms was higher among older adult diagnosed with chronic conditions (6). A systematic review and meta-analysis of 24 studies highlighted that depression was at approximately fourfold increased odds among frail older adults (7). The co-existence of depression and frailty leads to worse outcomes such as accelerated cognitive impairment, increased morbidity and mortality, and a declined QoL, emphasizing the need to explore potential modifiable factors associated with both conditions (7).

In this sense, proposed that physical activity (PA) may play a crucial role in promoting and preserving mental health by potentially mitigating the negative impacts of stress (8). According to the Physical Activity Guidelines for Americans, adults should perform at least 150 min to 300 min a week of moderate-intensity, or 75 min to 150 min a week of vigorous-intensity aerobic physical activity (9). A large-scale community-based study among 26,000 families in Saudi Arabia reported that only 17.4% of adults meet the minimum recommended physical activity levels and those aged ≥65 years old was the lowest practitioners of physical activity (10). The most common reason for not practicing physical activity was lack of desire (33.50%) (10). A recent literature review investigating the relationship between PA and depression revealed that there is an inverse relationship between PA and depression in older adults (11). However, none of the studies included in this review studied the association between sedentary behavior and depression. Further, the recommendation of this review mentioned that “reciprocal relationships between PA and depression in older adults still need more research to be replicated” (11).

Empirical evidence indicated that consistent engagement in PA, particularly moderate-to-vigorous physical activity (MVPA) (e.g., exercise, sports, and brisk walking) has been linked to reduced levels of depression in older adults (12). Similarly, a randomized control trial reported that multicomponent exercise program have a significant impact in reducing frailty (*p* < 0.001) and reversing frailty to pre-frailty in the older population (13).

Despite the known benefits of PA, little attention has been paid to the combined impact of frailty and depression on PA levels and QoL among older adults. Given the cultural and lifestyle differences in Saudi Arabia, it is essential to investigate these associations to addresses these modifiable factors and inform tailored interventions that can improve mental health, and frailty symptoms of older adults, ultimately enhancing their overall quality of life.

Therefore, the present study aimed to primarily examine the independent association of depression, frailty and their coexistence with PA levels among community-dwelling older adults in Saudi Arabia. The secondary aim was to investigate the independent association of depression, frailty and their coexistence with QoL among Saudi older adults. The results of the study may help healthcare professionals to understand the interplay association between coexisted frailty and depression and both PA and QoL, and thereby design an appropriate modifiable intervention to reduce their impact and improve overall QoL.

## Methods

### Study design

A cross-sectional study was conducted to examine the association between depression, frailty and coexisted frailty and depression and both PA level and QoL among community-dwelling older adults in Saudi Arabia.

### Data collection procedures

Adults’ community-dwellers were recruited to participate in the study. Participants were eligible if they were aged ≥ 60 years, able to read and write in Arabic, and were Saudi citizens. The data collection was conducted in-person by ten independent trained physiotherapy researchers (between March–September 2022). Many community locations were used for recruitment including malls, mosques, and social places.

Data were collected using a two-part, standardized form designed by the research team for the purpose of the study. The first part of the form contained questions related to demographics including age, gender, and body mass index (BMI). The second part of the form aimed to gather clinical data using standardized outcome measure including PA level, depression symptoms and QoL. All research assistants were trained on data collection. The validation process was taken place through randomly validating the results with some of the included participants through phone call. All data were collected via written questionnaires or interviews. The collected data were anonymized and confidentially protected. The recruited participants received a participant information sheet (includes study aim, procedure, their rights to withdraw anytime, and the assurance of the privacy of the collected data) and have an opportunity to ask anything about the study before participation. All participants signed an informed consent form in accordance with the Declaration of Helsinki. This study was approved by the Research Ethics Committee at Prince Sattam bin Abdulaziz University (No. RHPT/022/010).

### Outcome measures

#### Frailty

The Arabic version of the FRAIL scale is a 5-domain scale designed to evaluate physical frailty (14). These domains include resistance, fatigue, illness, ambulation, and weight loss. Scores on the scale range from 0 to 5, with 0 representing the best condition and 5 indicating the worst. The total FRAIL scale score is computed by summing up the scores from each of the five domains. A score of 3 to 5 indicates frailty, 1 to 2 indicates pre-frailty, and a score of 0 indicates no frailty. Arabic FRAIL scale demonstrated good diagnostic accuracy for frailty (AUC = 0.71) using the Fried Frailty Index as the criterion measure (14).

### Depressive symptoms

The Arabic version of the Patient Health Questionnaire 9 (PHQ-9) was used to assess depressive symptoms in this study (15). This questionnaire is a valid scale for detecting suspected cases of depression and anxiety in the general population as well as for assessing the severity of these mental diseases. PHQ-9 demonstrated a good reliability and validity in both original and Arabic versions (15, 16). The Arabic PHQ-9 showed good internal consistency with Cronbach’s alpha of 0.857 (15). All items in the Arabic version were correlated with the total scale to a good degree (lowest *r* = 0.378) (15).

PHQ-9 includes 9 questions related to depressive symptoms. These questions were used to investigate several domains of depression, including anhedonia, depressed mood, sleep problems, fatigue, change in appetite, decreased self-esteem, concentration disturbance, psychomotor disorder, and suicidal thoughts. Each question was rated on a 4-point scale (0–3): “not at all = 0,” “several days = 1,” “more than half the days = 2,” and “nearly every day = 3.” The total score of the PHQ-9 is the sum of the 9-item scores and ranges between 0 and 27. In the current study, participants were classified as having depressive symptoms when the PHQ-9 score was ≥10 (17, 18).

### Physical activity level

The Arabic version of Physical Activity Scale for the Elderly (PASE) was used to measure PA level (19, 20). PASE is a self-reported scale comprised of 12 items evaluates PA over a week focusing on participation in leisure activities, sports, and recreation, categorized by frequency and duration. Activities primarily involving a seated position, such as work (paid or unpaid), were recorded in total hours per week. Housework (light and heavy), lawn work/yard care, home repair, outdoor gardening, and caregiving for others were marked as dichotomous variables (“yes” or “no”). The total PASE score was calculated by summing all activities, with the time spent in each activity (hours/week) or participation in an activity (yes/no) multiplied by empirically derived item weights. The total PASE score range from 0 to 793; higher scores indicate greater PA. The PASE score was stratified in tertiles: 0 to 40 (sedentary), 41 to 90 (light physical activity) and more than 90 (moderate to intense activity) (21). The Arabic version of PASE demonstrated a good psychometric property. The internal consistency of the Arabic PASE components was good (Cronbach’s alpha 0.70–0.75), and the reliability of the components was excellent (ICC_2,1_ 0.90–0.98) (19).

### Quality of life

Health-related quality of life (QoL) was assessed using SF-12 (22). The SF-12 is a self-reported multipurpose measure of QoL derived from the SF-36 and contains only 12 items. The 12 items divided into two domains: Physical Component Summary (PCS) and the Mental Component Summary (MCS). The PCS focused on measuring the following QoL concepts: general health, mobility, physical activity limitations, stair climbing ability, and work limitations due to physical issues or pain. Whilest the MCS focuses on emotional aspects such as depression, anxiety, social activity, and the impact of feelings on productivity. The total QoL scores for PCS and MCS ranged from 0 to 100, where higher scores indicate better quality of life. The SF-12 have a good psychometric properties in different age groups, including older adults (23). The Arabic version have a good reliability with a high Cronbach’s alpha for the two components: MCS (*α* = 0.707) and PCS (*α* = 0.743) (24).

### Sample size calculation

The sample has been calculated based on previous evidence related to the prevalence of frailty in Saudi Arabia (1). The reported prevalence of frailty and pre-frailty was 21.4 and 47.3%. There is no study investigated the prevalence of depressive symptoms or a combination of frailty and depression in older adults in Saudi Arabia. Therefore, we calculated the sample size using this formula [*N* = Z^2^ P(1−P)/d^2^]. Where *N* = sample size, *Z* = Z score statistic for a level of confidence (1.96), *P* = the prevalence of frailty, and *d* = the degree of precision (0.05). The sample size was estimated to be 330. Therefore, we recruited 395 participants accounting for possible missing data.

### Statistical analysis

Descriptive statistics (mean, standard deviation, number, and percentage) were used to examine participants demographics and clinical characteristics. Differences between the groups (depression only, frailty only and coexisted frailty and depression) were determined by one-way ANOVA or Chi-square tests.

Multiple binomial logistic regression was utilized to examine the association between each group (depression only, frailty only and coexisted frailty and depression) and categories for PA level. Results are presented as odds ratios (OR) with 95% confidence intervals (95% CI) after controlling for covariates including age, sex, and BMI in this model.

Multiple linear regression was conducted to examine the association between each group (depressive only, frailty only and coexisted frailty and depression) and QOL. Two models were created, one for the PCS and the other model for the MCS after controlling for covariates including age, sex, and BMI. *p*-value was set at 0.05, and all the analyses used IBM SPSS for Mac version 29.0 (SPSS Inc. Chicago, IL).

## Results

### Participant characteristics

**Figure 1:**
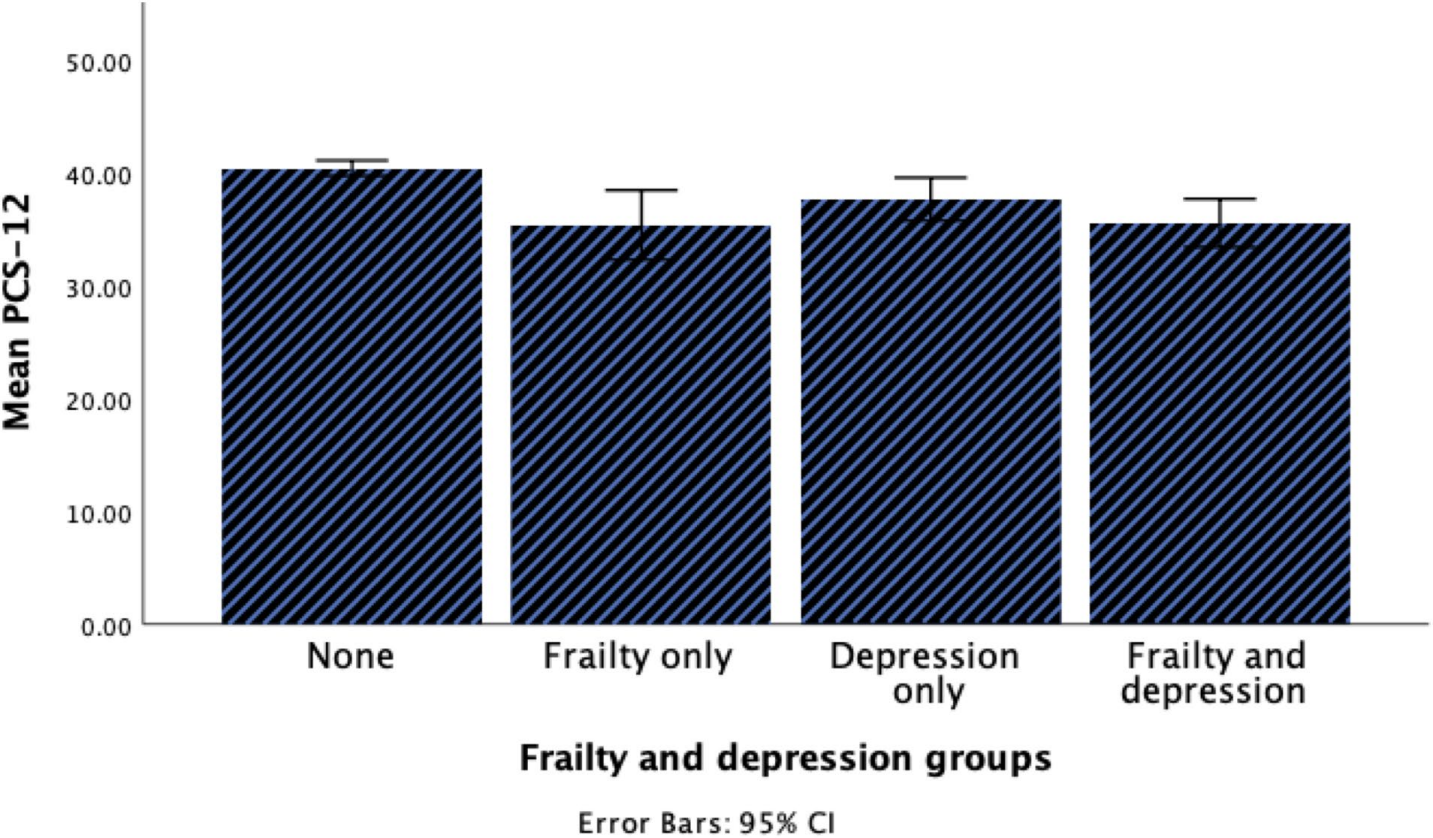
Histogram for the PCS of HRQOL among groups.

**Figure 2:**
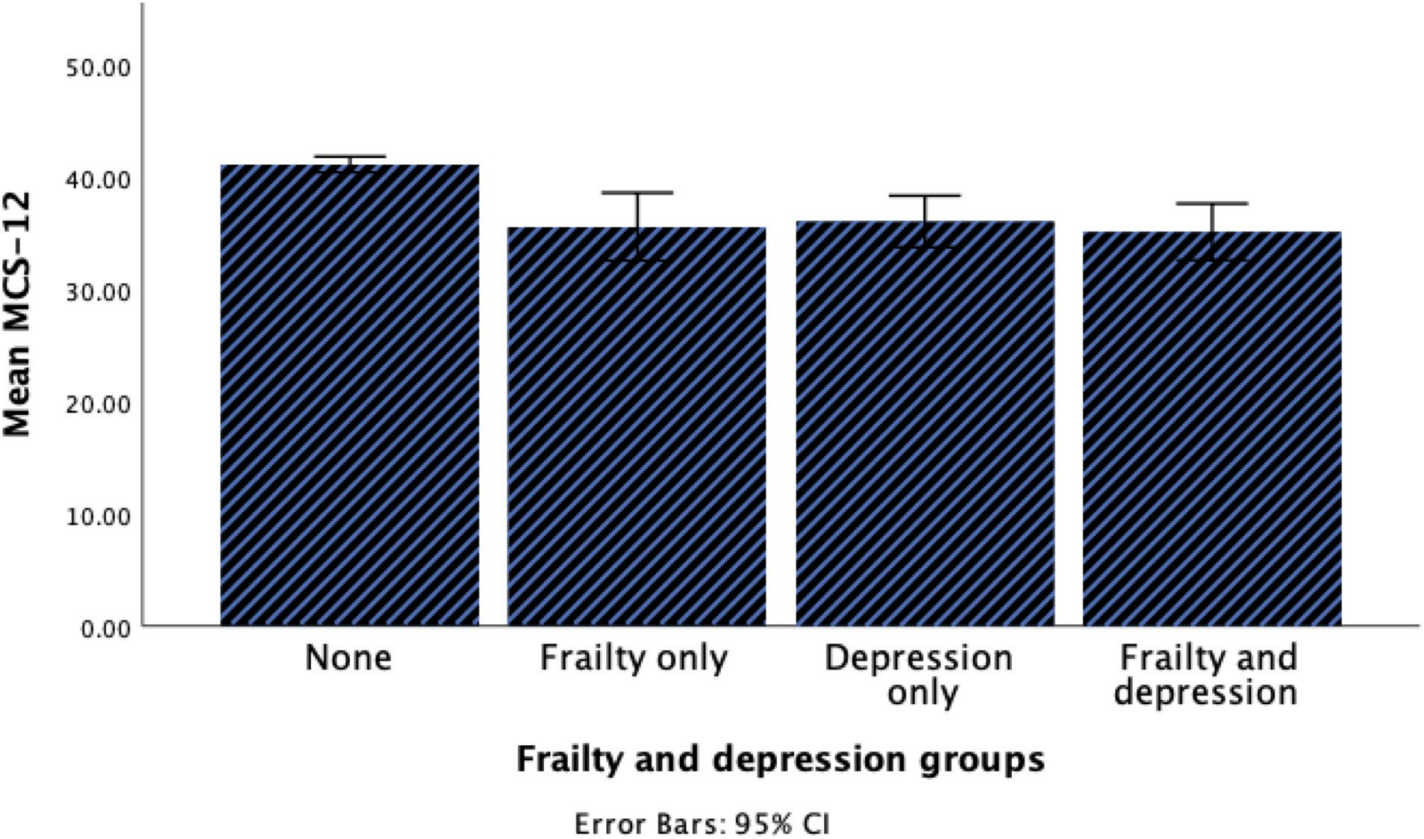
Histogram for the MCS of HRQOL among groups.

A total of 395 older adults participated in the current study. The demographic and clinical characteristics of the participants are summarized in Table 1. In brief, age, sex, quality of life, and level of PA were significantly different between groups. The prevalence of coexisting frailty and depression was 8.6% among participants. Participants with coexisting frailty and depression were notably older, and more than half of them exhibited a sedentary level of PA. Furthermore, coexisting frailty and depression group had lower scores in both PCS and MCS compared to other groups as illustrated in Figures 1, 2.

**Table 1:** Sociodemographic and clinical characteristics of the study sample.

Variables	Total sample *N* = 395	Coexisted frailty and depression *N* = 34 (8.6%)	Frailty only *N* = 28 (7.1%)	Depression only *N* = 43 (10.9%)	Neither frailty nor depression *N* = 290 (73.4%)	*p*-value
Age, years (mean ± SD)	66.4 ± 7.1	74 ± 8.3	69.6 ± 7	67.5 ± 7.8	65 ± 6.2	**0.003**
Sex, Female, *n* (%) within groups	238 (60.3)	22 (64.7)	19 (67.9)	36 (83.7)	161 (55.5)	**<0.001**
BMI (Kg/m^2^), (mean **±** SD)	28.7 ± 5.7	27.8 ± 5.5	31.3 ± 8.6	28.7 ± 6	28.6 ± 5.3	0.078
PASE categories *n* (%) within groups						
Sedentary	107 (27)	20 (58.8)	14 (50)	17 (39.5)	56 (19.3)	**<0.001**
Light physical activity	137 (34.7)	9 (26.5)	5 (17.9)	15 (34.9)	108 (37.2)	**<0.001**
Moderate to intense activity	151 (40.7)	5 (14.7)	9 (32.1)	11 (25.6)	126 (43.5)	**<0.001**
PCS of QOL (mean **±** SD)	39.4 ± 6.6	35.6 ± 6.1	35.4 ± 8	37.8 ± 6.8	40.5 ± 6	**<0.001**
MCS of QOL (mean **±** SD)	39.4 ± 7.1	35.1 ± 7.2	35.6 ± 7.8	36 ± 8	40.8 ± 6.4	**<0.001**

SD, Standard deviation.

Boldface *p*-value indicates a statistically significant difference using one-way ANOVA or Chi-square.

### Summary of main findings

This study examined the association between frailty, depression, and their coexistence with levels of physical activity and quality of life among community-dwelling older adults in Saudi Arabia. The Key findings reveal that those with co-existing frailty and depression were significantly more sedentary (OR = 4.37, 95% CI: 1.41–13.55, *p* = 0.011) having lower physical (PCS) and mental (MCS) quality-of-life scores compared with other groups. Depression and frailty independently contributed to lower quality of life. While physical component quality of life (PCS) had a more apparent association with frailty, the mental component (MCS) was more associated with depression.

### Association between groups and categories of physical activity level

Table 2 presents the results of multinomial logistic regression examining the association between frailty, depression, and coexisting frailty and depression with different levels of PA (PASE categories). The regression model has been adjusted for potential confounding factors, including age, sex, and BMI.

**Table 2:** Multinomial logistic regression for the association between each group with level of physical activity (PASE).

Groups	Activity level (PASE)	OR (95% CI)	*p-*value
Depression only	Sedentary	3.08 (1.26, 7.55)	**0.014**
	Light physical activity	1.5 (0.65, 3.48)	0.342
Frailty only	Sedentary	1.92 (0.7, 5.25)	0.201
	Light physical activity	0.53 (0.17, 1.66)	0.276
Coexisted frailty and depression	Sedentary	4.37 (1.41, 13.55)	**0.011**
	Light physical activity	1.64 (0.51, 5.27)	0.406

OR, Odds ratio.

CI, Confidence interval.

Boldface *p*-value indicates a statistically significant difference.

The reference category is: Neither frailty nor depression.

The results demonstrate a significant association between PA levels and the presence of depression or a coexistence of depression and frailty. Participants with depressive symptoms were significantly more likely to be sedentary (OR = 3.08, 95% CI: 1.26–7.55, *p* = 0.014) compared to those without frailty or depression. Additionally, coexisted frailty and depression group showed a significant association with PA level, and participants in this group were more likely to be sedentary (OR = 4.37, 95% CI: 1.41–13.55, *p* = 0.011). However, frailty alone showed no significant associations (*p* > 0.05) with sedentary or light PA levels.

### Association between groups and quality of life

Tables 3, 4 presents the results of the multiple linear regression examining the association between frailty, depression, and coexisting frailty and depression with physical (PCS) and mental (MCS) subscales and of quality of life. The regression models have been adjusted for potential confounding factors, including age, sex, and BMI.

**Table 3:** Multiple linear regression for the association between each group with PCS of the quality of life (SF12).

Groups	*B*	SE	*p-*value
Depression only	− 1.69	0.96	0.133
Frailty only	− 3.55	1.2	**0.003**
Coexisted frailty and depression	− 2.55	1.2	**0.033**
*R*^2^ = 0.14			

SE, Standard error.

Boldface *p*-value indicates a statistically significant difference.

**Table 4:** Multiple linear regression for the association between each group with MCS of the quality of life (SF12).

Groups	*B*	SE	*p-*value
Depression only	− 4.81	1.1	**<0.001**
Frailty only	− 4.67	1.3	**<0.001**
Coexisted frailty and depression	− 4.76	1.2	**<0.001**
*R*^2^ = 0.12			

SE, Standard error.

Boldface *p*-value indicates a statistically significant difference.

Both groups, the frailty only and the coexisting frailty and depression group, were associated with lower values in the physical component summary (PCS) as shown in Table 3. The association results indicated a negative association of frailty with the physical quality of life (PCS) (*B* = −3.65, SE = 1.2, *p* = 0.003). Further, the association results suggested that coexisting frailty and depression affect the PCS score negatively (B = −2.53, SE = 1.2, *p* = 0.033). However, depression alone showed no significant associations with PCS score (*p* = 0.133), suggesting that depression independently may not significantly affect the physical domain of QoL.

For the mental component summary (MCS), all groups (frailty, depression, and coexisting frailty and depression) were significantly negatively associated with MCS scores (*p* < 0.001) as shown in Table 4. Frailty only group was significantly negatively associated with MCS score (*B* = −4.67, SE = 1.3, *p* < 0.001), indicating that frailty has a negative influence on mental well-being. Similarly, depression only group had the greatest negative association with MCS score (*B* = −4.81, SE = 1.1, *p* < 0.001), suggesting that depression is a major factor contributing to poor mental QoL. In addition, the frailty and depression coexisting were negatively associated with MCS score (*B* = −4.76, SE = 1.2, *p* < 0.001), emphasizing the role of both conditions in the deterioration of mental QoL.

Overall, frailty mainly affects the physical component of QoL; whilest depression has a stronger negative impact on mental component of QoL. The coexisting frailty and depression have a negative effect on both physical and mental quality of life.

## Discussion

### Main findings

This study aimed to explore the association between frailty, depression, or their coexistence with PA level. Further, it investigated the independent association between depression, frailty and coexisted frailty and depression with physical and mental quality of life among older adults. The findings revealed that a significant decline in PA level in depression and coexisting frailty and depression group as both of them are significantly associated with higher likelihood of being sedentary. Additionally, frailty alone and coexisting frailty and depression significantly reduce the physical aspect of quality of life (PCS). in the other hand, both frailty and depression, whether independently or coexisting, significantly reduce the mental aspect of quality of life (MCS).

### Comparison with existing literature

The current study findings align with recent research that demonstrated a significant association between PA level as measured by total PASE score and depression (r_s_ = −0.371, *p* < 0.001) in older adults in Taiwan (25). The association between PA level and depression could be bidirectional as shown in a large cross-sectional study that analysed data from 14 European countries (26). A data from 32,392 European late middle-aged to older adults showed that moderate and vigorous PA at least once a week is negatively associated with the score of depression (*p* < 0.001) (26). In addition, results from a recent cross-sectional study conducted in China among 1,180 older adults (aged ≥ 60 years) found that lower PA levels were significantly associated with depressive symptoms among older women (*B* = 1.35, *p* < 0.001) (27).

On the other hand, frailty alone was not significantly associated with the PA levels in our findings, although previous studies conducted in Brazil and Taiwan shown that frailty associated with low levels of PA (25, 28). In Brazil, a longitudinal study evaluated the relationship between physical activity levels, sedentary behavior, and frailty among older adults found that low PA levels and excessive sedentary behavior (≥540 min per day) were associated with a higher prevalence of frailty (PR = 2.83, *p =* 0.01) (28). In Taiwan, the result of a cross-sectional study investigated the associations between physical activity and frailty indicated that frailty associated (r_s_ = −0.423, *p* < 0.001) with the total PASE score in older adult population (25). This disagreement between studies and our findings could be raised from the differences in the prevalence of frailty in the study’s population as only 7.1% of our sample have frailty, whereas frailty was more prevalent in the Taiwanese study sample, affecting 22.6% of participants (25).

Robust associations were found between various QoL domains (PCS and MCS) and frailty, as well as coexisting frailty and depression. However, depression alone showed a significant negative association only with the mental domain of QoL. This finding aligns with a previous literature that reported a negative correlation between frailty and QoL (4, 29–31). A systematic review and meta-analysis investigating the relationship between frailty and QoL among community-dwelling older adults revealed significantly higher PCS score on the SF-36 scale in non-frail group compared to those with frailty (30). Furthermore, a cross-sectional study used a multinomial logistic analysis of 573 individuals aged over 65 years old and lived in Spain identified the factors associated with QoL in community-dwelling older adults (29). Their results noted that frailty was the most strongly associated factor with diminished QoL, significantly affecting both the MCS and PCS. The odds of older adults having a low PCS were seven times greater for those who were frail (OR = 7.43, 95% CI = 2.13–25.82) compared to those who were robust. Similarly, the odds of having a low MCS were three times greater for those who were frail (OR = 3.2, 95% CI = 1.21–8.46) compared to those who were robust.

On the other hand, our finding is partially consistent with previous literature investigating the association between depression and QoL (32, 33). A systematic review and meta-analysis of longitudinal studies investigating the longitudinal association between depression and QoL showed that individuals with baseline depressive disorders had significantly (*p* < 0.01) lower PCS and MCS scores at follow-up compared to those without baseline depression, with medium and large effect sizes, respectively (30). Additionally, recent research among older adults in Greece suggested a significant independent association between frailty and depression with the physical domain of QoL (32). Their results indicated a decline in the physical domain of QoL as frailty and depression increased. However, they found that the mental domain of QoL was associated with depression scores but not with frailty. The differences between our findings and previous research could be due to the different scales used to measure depression, frailty, and QoL (32). Furthermore, the age of the sample in previous studies included younger participants (age range: 18–65) which could affect the prevalence of frailty and depression among participants (32, 33).

### The interrelationships between frailty, depression, physical activity, and quality of life

The findings of our study align with the theoretical framework that conceptualize frailty and depression as interacting geriatric syndromes that are influenced by overlapping common biological and functional pathways (34, 35).

The negative association between coexisting frailty and depression with the sedentary level of PA is not surprising given the overlap of frailty characteristics, depressive symptoms with reduction in PA. Frailty and depression are among the most strongly comorbid illness; with approximately 40% of frail older adults having depression and approximately 40% of people with late-life depression are frail (7). This bidirectional association between frailty and depression highlighted in two landmark studies by Fried and colleagues and Lakey and colleagues (3, 35). Fried and colleagues found a significant difference (*p* < 0.001) in depression among alder adults with 31% of frail older adults have depression compared to 3% of non-frail elders (3).

Using data from the Women’s Health Initiative Observational Study (*n* = 33,324), Lakey and colleagues reported that non-frail women with high depressive symptom scores were 2.19 times likely to become frail over 3 years than women without depressive symptoms (35).

These cross-sectional associations are supported by longitudinal studies further demonstrating this bidirectional risk (36, 37). A longitudinal study among 2,717 older adults found that older adults with depressive symptoms are more likely to develop frailty than nondepressed individuals (2.6 increased hazard) over an average follow-up period of 5.9 years (36). Conversely, a large scale (*n* = 25,771 older adults) 5-years longitudinal study found that pre-frail older adults had an 1.86 higher odds of depression, and frail older adult had more than twice the odds (OR = 2.46) compared to non-frail individuals (37).

Our study expands upon the previous literature by demonstrating that the coexisting frailty and depression associated with the decline in PA levels and reduction in QoL more than either condition alone. The interrelationship between frailty, depression, physical activity, and quality of life in older adults has been documented in previous literature (38, 39). A cross-sectional study explored the mediating effects of daily physical activity on the relationship between frailty and QoL in Taiwanese older adults (39). Their results demonstrate that daily physical activity mediated the relationship between frailty and QoL and higher physical activity correlated with lower frailty scores and better QoL. More recently, a cross-sectional study involving 235 pre-frail/frail older adults explored the interplay between frailty, physical function, physical activity and their impact on the QoL and depressive status in older adults with frailty (38). The findings revealed a significant correlation between QoL and aspects of physical frailty. Better QoL score was associated with low fatigue, high gait speed and higher engagement in light physical activity. Furthermore, worsening depressive symptoms was associated with slow gait speed and low level of physical activity. Their findings suggested that physical activity is a key contributor to improve quality of life and reduce depressive symptoms in older adults with frailty.

### Proposed mechanisms

The overlap between depression and frailty could be explained by the shared biological mechanisms that result in increased vulnerability and negative health outcomes in older adults. Brown et al. proposed that late life depression and frailty is the result results from shared biological mechanisms, including mitochondrial dysfunction, dopamine dysregulation, and chronic inflammation (34). Changes in mitochondrial function are apparent with age and characterized by reduced adenosine triphosphate (ATP) production, leading to fatigue, mobility decline, and an increase in oxidative stress. This mitochondrial dysfunction has been identified to be linked with reduced activity levels and depression. Another possible mechanism was dopamine dysfunction, particularly diminished levels of D1/D2 receptors in both the caudate and the putamen in older adults. This decrease is associated with decreased motor speed and worsening in frontal functioning, thereby exacerbate both conditions. Chronic inflammation, with raised levels of pro-inflammatory cytokines such as IL-6 and TNF-*α*, and C-reactive protein (CRP), affect the central nervous system—specifically, dopaminergic function in the basal ganglia, which may result in depression, fatigue, and cognitive and motor slowing. Chronic inflammatory state contributes to specific characteristics of frailty including low energy, decreased muscle mass and strength, cognitive impairments, reinforcing the bidirectional relationship between depression and frailty (34).

### Clinical implications

The strong association between coexistence of frailty and depression with the sedentary PA level and reduced QoL among community-dwelling older adults have significant clinical implications. The complexity of health issues among older adults especially with the coexistent frailty and depression implies that healthcare providers must adopt a multidisciplinary approach in managing these population. In routine geriatric assessment, regular evaluation of frailty and depressive symptoms, physical activity levels, and quality of life is necessary to inform the design of targeted interventions. A collaborative multidisciplinary program that addresses both mental and physical health is recommended. These may include mental health support (e.g., cognitive-behavioral therapy) to help in addressing the psychological barriers to physical activity and reduce sedentary behavior in this population. Engaging in regular physical activity program including aerobic, resistance, balance, and flexibility exercises plays a beneficial role in reducing frailty, enhancing physical function, decreasing psychological distress, and improving overall mental well-being (8, 13, 39).

In addition, health education could help in raising the awareness about the symptoms of frailty and depression and how physical exercise could mitigate their impact. Furthermore, motivational strategies and lifestyle interventions to enhance physical active lifestyle are recommended to promote a healthy aging, reduce the impact of frailty and depression, and thereby improve the quality of life.

### Study contributions and limitations

This study is one of the first to specifically examine the coexistence of frailty and depression and their combined impact on PA and QoL in older adults. The study contributes to the existing literature by quantifying the associations between frailty, depression, their coexistence with the PA levels and QoL in an older adult Saudi Arabian population. The results highlights the importance of developing a multidisciplinary interventions to address the impact of both frailty and depression and emphasize the QoL of older adults. Nevertheless, several limitations of this study should be acknowledged. First, the assessment of PA, depressive symptoms and QoL relied on self-reported measures, which may be subject to reporting bias. Second, the sample was recruited from a specific region of Saudi Arabia, which may limit the generalizability of results to other populations or cultural contexts. Further, the cross-sectional design of this study limits the ability to infer causality between frailty, depression, their coexistence, and the lower scores of QoL or PA.

## Conclusion

The novel contribution of this study is the focus on the combined impact of the coexisting frailty and depression on physical activity and quality of life among Saudi older adults. This study demonstrates a significant association between depression and coexisting frailty and depression with the sedentary level of PA among older adults. Individuals with coexisting frailty and depression were over four times more likely to be sedentary compared to those without either condition (OR = 4.37, 95% CI: 1.41–13.55). Furthermore, frailty, depression, their coexistence significantly associated with a decline in MCS of QoL. The lower score of MCS was associated with the frailty and coexisting frailty and depression groups. Individuals with coexisting frailty and depression had a 2.55-point decrease in MCS score compared to those without either condition (*B* = −2.55, *p* = 0.033). These findings highlight the need for multidimensional geriatric assessments including frailty and depressive symptoms evaluation to identifying modifiable risk factors associated with low levels of PA and QoL. Designing of a targeted interventions should be considered by multidisciplinary healthcare team (i.e., medical doctors and physiotherapists) to reduce the influence of frailty and depression in older adults and improve QoL. Moreover, public health policies should support the implementation of motivational strategies to raise an awareness about the impact of coexisting frailty and depression, promote a healthy lifestyle and address the barriers to physical activity.

While this study provides valuable insights, it is important to acknowledge its limitations, including its cross-sectional nature. Further research is required to establish causality through longitudinal studies that provide deeper insights into how coexisting frailty and depression influence PA and QoL over time in older adults. Furthermore, inclusion of additional measures such as Basal Metabolic Rate (BMR) and biomarkers (e.g., cortisol) might assist in gaining deeper insights into the physiological mechanisms underpinning the coexisting frailty and depression, and their effect on physical activity levels.

## Data Availability

The raw data supporting the conclusions of this article will be made available by the authors, without undue reservation.
